# Machine learning-based identification of anxiety symptoms in Chinese community-dwelling older adults: a comparative study of six algorithms with SHAP analysis and nomogram development

**DOI:** 10.3389/fpsyt.2026.1874650

**Published:** 2026-08-20

**Authors:** Peiyue Li, Chunxiao Yan, Jianbo Li, Qimi Zheng, Huiqi Zhang

**Affiliations:** 1 School of Nursing, Dazhou Vocational and Technical College, Dazhou, Sichuan, China; 2 Geriatrics Department, Dazhou Central Hospital, Dazhou, Sichuan, China; 3 Department of General Practice and Geriatrics, Dazhou Third People's Hospital Dachuan District People's Hospital, Dazhou, Sichuan, China

**Keywords:** anxiety symptoms, China, machine learning, nomogram, older adults, risk identification model

## Abstract

**Background:**

Anxiety disorders are common among older adults but remain underrecognized in community settings, particularly in China where mental health resources are scarce. This study aimed to develop and compare multiple machine learning models for identifying anxiety symptoms in Chinese community-dwelling older adults and to construct a practical clinical tool.

**Methods:**

A cross-sectional study included 5,331 community-dwelling older adults (aged ≥65 years) from Shenzhen, China. Anxiety symptoms were assessed using the Generalized Anxiety Disorder-7 (GAD-7) scale, with a score ≥5 indicating clinically significant anxiety. Six machine learning algorithms—Lasso, Random Forest, XGBoost, Decision Tree, Support Vector Machine, and Gradient Boosting Machine (GBM)—were trained and compared. Model performance was evaluated using area under the curve (AUC), sensitivity, specificity, and accuracy. SHapley Additive exPlanations (SHAP) was employed for model interpretability, and a nomogram was developed for clinical application.

**Results:**

The prevalence of anxiety symptoms was 11.3% (605/5,331). GBM achieved the highest AUC of 0.900 (sensitivity = 0.890, specificity = 0.762), comparable to XGBoost (AUC = 0.897) and Lasso (AUC = 0.893) with no significant differences (all P > 0.05). Feature combination analysis revealed that the “Psychological + Clinical” set achieved optimal performance (AUC = 0.903, PR-AUC = 0.617). SHAP analysis identified depressive symptoms (PHQ-9), insomnia (ISI), and loneliness (ULS-6) as the top three risk indicators. A nomogram incorporating nine predictors demonstrated good clinical utility.

**Conclusions:**

Machine learning models, particularly GBM, showed excellent performance in identifying anxiety symptoms among Chinese community-dwelling older adults. The model demonstrates promising internal validity but remains internally validated only; clinical implementation is premature without external validation in independent cohorts.

## 1 Introduction

Anxiety disorders are among the most prevalent mental health conditions worldwide, affecting approximately 4.05% of the global population (1). In China, the rapid aging of the population has brought mental health issues among the elderly to the forefront of public health concerns. According to the China Mental Health Survey (CMHS), the 12-month prevalence of anxiety disorders among Chinese older adults aged 55 years and above is 5.97%, with a lifetime prevalence of 9.07% (2). Despite this high burden, the mental health service gap for anxiety disorders among Chinese older adults is alarming—only 11.8% of affected individuals seek any mental health services (3). Anxiety in older adults is often underrecognized and undertreated, as symptoms are frequently misattributed to normal aging or physical illness (4, 5). Moreover, the traditional reliance on clinical interviews and self-report questionnaires, while valuable, has inherent limitations in identifying high-risk individuals, particularly in community settings where mental health resources are scarce (6). The clinical reality is that simple, non-invasive, and scalable screening tools that can be administered by primary care providers without specialized psychiatric training remain urgently needed (7, 8).

Machine learning (ML) offers a promising approach to mental health screening by capturing complex, non-linear relationships among multiple risk factors and automating risk stratification. Previous machine learning (ML) studies on anxiety identification have several critical limitations. First, most ML research in mental health has focused on depression rather than anxiety specifically, leaving anxiety identification comparatively underexplored (9, 10). Second, the majority of existing ML-based anxiety identification studies have been conducted in Western populations (e.g., Canadian Longitudinal Study on Aging (11), and their generalizability to Chinese older adults is uncertain given substantial differences in cultural contexts, healthcare systems, and aging experiences (2, 12). Third, prior studies often employed single algorithms without systematic comparison, and many lacked model interpretability features—a crucial gap, as healthcare providers cannot be expected to trust or act upon “black-box” predictions without understanding what drives them (13, 14). Fourth, existing models have frequently relied on costly or invasive biomarkers [e.g., heart rate variability (15)] or disease-specific samples [e.g., COPD or abdominal obesity patients (16, 17)], limiting their utility for routine community-based screening. To address the interpretability gap, SHapley Additive exPlanations (SHAP) has emerged as a unified framework for explaining ML model predictions by quantifying each feature’s contribution (18). Furthermore, translating complex ML models into practical clinical tools—such as nomograms and risk score sheets—is essential for bridging the gap between computational psychiatry and frontline clinical practice (19, 20).

Therefore, this study aimed to address these gaps by (1): developing and systematically comparing multiple machine learning algorithms to identify anxiety symptoms in community-dwelling older adults (2); identifying key risk factors using SHAP analysis to enhance model interpretability; and (3) developing a practical logistic regression-based nomogram and point-based risk score sheet for clinical application. By leveraging only simple, self-reported questionnaire data that can be collected in routine primary care visits, our approach offers a scalable, non-invasive, and clinically actionable tool for early anxiety detection in community settings. The significance of this work lies in its potential to reduce the mental health service gap for anxious older adults in China, enabling timely intervention and potentially preventing the progression to more severe anxiety disorders and associated functional impairment.

## 2 Materials and methods

### 2.1 Study design and participants

This cross-sectional study conducted a secondary analysis of de-identified data from the Shenzhen Mental Health Survey of the Elderly (SMHSE), a community-based survey of adults aged ≥65 years in Shenzhen, China. The original survey employed multi-stage stratified cluster sampling to recruit participants from residential communities, with trained interviewers administering structured questionnaires via face-to-face interviews. The SMHSE was approved by the Research Ethics Committee of Shenzhen People’s Hospital, and all participants provided written informed consent for data collection and secondary research use at the time of enrollment (21).

**Figure 1:**
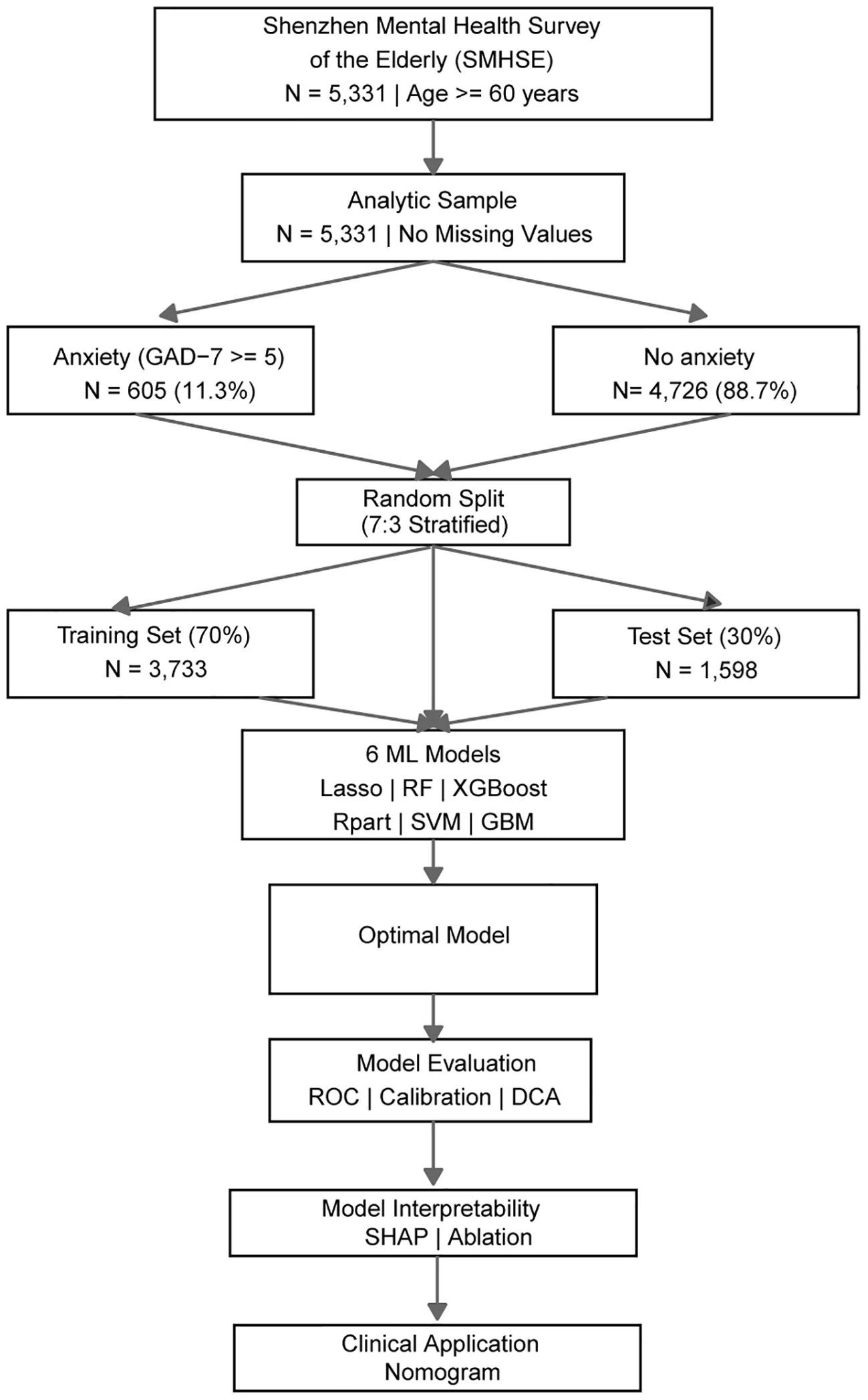
Flow diagram of the study design and data processing. SMHSE, Shenzhen Mental Health Survey of the Elderly; GAD-7, Generalized Anxiety Disorder 7-item scale; ML, machine learning; GBM, Gradient Boosting Machine; SHAP, SHapley Additive exPlanations.

Of 5331 participants with complete data, 605 (11.3%) met criteria for clinically significant anxiety symptoms (GAD-7 ≥ 5) (22, 23). The sample was randomly split into training (70%, n = 3733) and testing (30%, n = 1598) sets using stratified sampling to preserve outcome distribution (21).The overall study flow diagram is presented in **Figure 1**.

### 2.2 Measurements

#### 2.2.1 Outcome

Anxiety symptoms were assessed with the Generalized Anxiety Disorder-7 (GAD-7), a 7-item scale scored 0–21. A cutoff of ≥5 defined clinically significant anxiety symptoms. This cutoff has been widely used to identify clinically significant anxiety symptoms in community-based older adult populations (22, 23). The Cronbach’s alpha for the GAD-7 in this sample was 0.911 (21).

#### 2.2.2 Predictors

Sixteen variables were selected based on a systematic review of established risk factors for late-life anxiety and data availability in the SMHSE dataset (21), encompassing five domains: sociodemographic characteristics, health behaviors, psychological scales, clinical conditions, and sleep (21):

#### 2.2.3 Psychological scales

PHQ-9 (depression, 0–27, cutoff ≥5 for depressive symptoms, α=0.805); ISI (insomnia, 0–28, cutoff ≥7 for insomnia symptoms, α=0.929); ULS-6 (loneliness, 6–24, α=0.870); AD-8 (cognitive decline, 0–8, cutoff ≥2 for MCI (Mild Cognitive Impairment), α=0.796); CSI-D (dementia screening, 0–9, cutoff ≤7 for early dementia, α=0.674).

#### 2.2.4 Sleep

Self-reported sleep duration (hours/night).

#### 2.2.5 Sociodemographics

Education (4 levels: primary or below/junior high or senior high/college/postgraduate), marital status (2 levels: married/cohabiting vs. unmarried/divorced/widowed), monthly income (4 levels: <1000/1000-2999/3000-4999/≥5000 RMB).

#### 2.2.6 Health behaviors

Smoking (3 levels: never/former/current), drinking (3 levels: never/former/current), self-rated health (4 levels: excellent/good/fair/poor).

#### 2.2.7 Clinical conditions

Number of chronic diseases (5 levels: 0/1/2/3/≥4), insomnia diagnosis (yes/no), MCI (yes/no), early dementia (yes/no).

All scales used validated Chinese versions. The SMHSE dataset contained complete data for all 5,331 participants on all variables included in this analysis; therefore, no participants were excluded due to missing data, no imputation was required, and a complete-case analysis was effectively performed. The original survey protocol ensured data completeness through in-person interview administration with immediate verification by field investigators (21).

### 2.3 Statistical analysis

Given the cross-sectional design, our models identify concurrent anxiety status rather than predict future onset of anxiety. All statistical analyses were conducted using R version 4.1.0 (R Foundation for Statistical Computing, Vienna, Austria). Continuous variables were presented as mean ± standard deviation (SD). Between-group comparisons for psychometric scale scores were conducted using the Mann-Whitney U test due to non-normal distributions; normally distributed variables were compared using independent samples t-tests. Categorical variables were compared using chi-square tests. A two-tailed P-value < 0.05 was considered statistically significant.

### 2.4 Model development and comparison

The dataset was randomly split into training (70%, n = 3,733) and test sets (30%, n = 1,598) using stratified sampling with a fixed random seed [set.seed (42) in R] to ensure reproducibility of the split. No oversampling technique (e.g., SMOTE) was applied to the training set, as we aimed to evaluate model performance under the natural class distribution (anxiety prevalence: 11.3%). While a prevalence of 11.3% is not extremely imbalanced (typically defined as <5% or >90%), we chose not to apply SMOTE for three reasons (1): the imbalance ratio (1:8) is modest and tree-based methods (Random Forest, GBM, XGBoost) are relatively robust to moderate imbalance (2); SMOTE can introduce synthetic samples that may not reflect real-world data distributions, potentially biasing performance estimates; and (3) our primary interest was in real-world screening performance, where the natural prevalence must be respected. To assess the potential impact of imbalance, we performed threshold adjustment (**Table 1**) and report PR-AUC alongside standard AUC, as PR-AUC is more informative for imbalanced settings.

**Table 1:** Model performance at different probability thresholds for anxiety identification.

Threshold	Sensitivity	Specificity	PPV	NPV
0.03	0.934	0.629	0.244	0.987
0.05	0.884	0.761	0.321	0.981
0.07	0.823	0.808	0.354	0.973
0.10	0.746	0.856	0.398	0.963

PPV, positive predictive value; NPV, negative predictive value.

Six machine learning algorithms were developed: Lasso, Random Forest, XGBoost, Decision Tree (Rpart), Support Vector Machine, and Gradient Boosting Machine (GBM), implemented in R using glmnet, randomForest, xgboost, rpart, e1071, and gbm packages. Hyperparameters were optimized via grid search with 5-fold cross-validation on the training set. For algorithms with stochastic elements (Random Forest, GBM, XGBoost), random seeds were fixed [set.seed (123) for model training] to ensure reproducibility. Model performance was evaluated using AUC, accuracy, sensitivity, specificity, PPV, NPV, and F1 score. The optimal threshold was determined by Youden’s J index. Calibration curves and decision curve analysis assessed model calibration and clinical utility. The DeLong test compared AUC differences between models. The best-performing model was selected for SHAP analysis, subgroup analyses, and nomogram development.

### 2.5 Model interpretability

To address the “black-box” nature of machine learning models, SHAP (SHapley Additive exPlanations) was employed to interpret the outputs of the optimal GBM model. SHAP values were calculated on the test set (n = 1,598) using the trained GBM model, ensuring that interpretation was based on unseen data to avoid overfitting bias. This method, grounded in cooperative game theory, assigns each feature a Shapley value representing its marginal contribution to the model’s output. Features with larger absolute Shapley values exert greater influence on the identification and are therefore considered more important. The sign of the SHAP value indicates the direction of the effect: positive values drive the prediction toward higher anxiety risk, whereas negative values push the prediction toward lower risk. Visualization of SHAP values, including bar plots for global feature importance and beeswarm plots for effect direction, was performed using the shapviz package in R.

### 2.6 Sensitivity and subgroup analyses

To assess model robustness, sensitivity analyses were conducted from two perspectives. First, we compared model performance with and without SMOTE balancing to evaluate the impact of class imbalance given the 11.3% prevalence of anxiety. Second, an ablation study sequentially removed the top five SHAP-identified features, with AUC declines quantified via 1,000 bootstrap resamples to validate their necessity.

Subgroup analyses evaluated model generalizability across clinically relevant strata in the test set (n = 1,598). Stratification factors included educational attainment (primary or below vs. junior high and above), marital status (with vs. without spouse), self-perceived health (good vs. poor), multimorbidity (0–1 vs. ≥2 chronic conditions), clinical insomnia (present vs. absent), and comorbid depressive symptoms (PHQ-9 ≤5 vs. >5). Discriminative performance for each subgroup was measured by AUC with 95% confidence intervals.

### 2.7 Nomogram application

To facilitate clinical application, a nomogram was constructed using standard logistic regression based on the top five predictors identified by SHAP analysis (PHQ-9, ISI, ULS-6, AD-8, and sleep duration). The logistic regression model was fitted on the training set using the rms package in R, and the nomogram was derived from the regression coefficients. For an individual patient, points are assigned to each predictor based on their value; the total points are summed and converted to an estimated probability of anxiety symptoms. Additionally, a simplified point-based risk score sheet was derived from the nomogram, allowing frontline healthcare providers to quickly stratify patients into low-, moderate-, or high-risk groups without computational tools. The nomogram is accessible as a digital version and can be easily integrated into routine primary care workflows for anxiety screening in older adults.

## 3 Results

### 3.1 Participant characteristics

**Table 2** compares the baseline characteristics between the training set (n = 3,733) and test set (n = 1,598). The results show that the mean age was 68.5 ± 6.8 years (mean ± SD) in the training set and 68.3 ± 6.9 years in the test set, with no statistically significant difference (P = 0.845). The proportion of female participants was 56.1% in the training set and 55.9% in the test set (P = 0.934).

**Table 2:** Comparison of baseline characteristics between training and test sets.

Variable	Training (n = 3,733)	Testing (n = 1,598)	P value
Education, n (%)			0.486
Primary or below	1,585 (42.5)	645 (40.4)	
Junior high	948 (25.4)	438 (27.4)	
Senior high	728 (19.5)	303 (19.0)	
College/University or above	472 (12.6)	212 (13.3)	
Marital status, n (%)			0.425
Married/Cohabiting	702 (18.8)	285 (17.8)	
Unmarried/Divorced/Widowed	3,031 (81.2)	1,313 (82.2)	
Monthly income, n (%)			0.119
<1000 RMB	1,509 (40.4)	585 (36.6)	
1000–2999 RMB	691 (18.5)	326 (20.4)	
3000–4999 RMB	830 (22.2)	375 (23.5)	
≥5000 RMB	703 (18.8)	312 (19.6)	
Chronic diseases, n (%)			0.047
No	1,248 (33.4)	489 (30.6)	
Yes	2,485 (66.6)	1,109 (69.4)	
Self-rated health, n (%)			0.174
Excellent	557 (14.9)	244 (15.3)	
Good	1,225 (32.8)	514 (32.2)	
Fair	1,570 (42.1)	654 (40.9)	
Poor	381 (10.3)	186 (11.6)	
Drinking, n (%)			0.146
Never	2,896 (77.6)	1,231 (77.0)	
Former	294 (7.9)	150 (9.4)	
Current	543 (14.5)	217 (13.6)	
Smoking, n (%)			0.179
Never	2,960 (79.3)	1,235 (77.3)	
Former	337 (9.0)	168 (10.5)	
Current	436 (11.7)	195 (12.2)	
Insomnia, n (%)			0.761
No	2,996 (80.3)	1,276 (79.8)	
Yes	737 (19.7)	322 (20.2)	
MCI, n (%)			0.451
No	3,002 (80.4)	1,300 (81.4)	
Yes	731 (19.6)	298 (18.6)	
Early dementia, n (%)			0.822
No	2,915 (78.1)	1,253 (78.4)	
Yes	818 (21.9)	345 (21.6)	
Psychological scales (mean ± SD)			
PHQ-9	1.23 ± 3.24	1.09 ± 2.84	0.126
GAD-7	1.40 ± 2.86	1.33 ± 2.59	0.447
ISI	3.92 ± 4.89	4.06 ± 4.85	0.368
ULS-6	7.54 ± 2.81	7.46 ± 2.75	0.344
Sleep duration (hours)	6.94 ± 1.76	6.89 ± 1.73	0.269
AD-8	0.99 ± 1.71	0.96 ± 1.61	0.457
CSI-D	8.22 ± 1.25	8.26 ± 1.17	0.205
Anxiety symptoms, n (%)			1.000
No	3,309 (88.6)	1,417 (88.7)	
Yes	424 (11.4)	181 (11.3)	

P values were calculated using chi-square tests for categorical variables and Mann-Whitney U tests for continuous variables. MCI, Mild Cognitive Impairment; CSI-D, Community Screening Instrument for Dementia.

Regarding psychological scales, the training and test sets showed comparable scores on PHQ-9 (1.23 ± 3.24 vs. 1.09 ± 2.84, P = 0.126), ISI (3.92 ± 4.89 vs. 4.06 ± 4.85, P = 0.368), and ULS-6 (7.54 ± 2.81 vs. 7.46 ± 2.75, P = 0.344). The prevalence of anxiety symptoms (GAD-7 ≥ 5) was nearly identical between the two sets (11.4% vs. 11.3%, P = 1.000).

For categorical variables, no significant differences were observed across education level (P = 0.486), marital status (P = 0.425), self-rated health (P = 0.174), smoking (P = 0.179), or drinking (P = 0.146). The only exception was chronic diseases (P = 0.047), though the difference was marginal. Overall, the training and test sets were well-balanced across all demographic, clinical, and psychological variables, confirming the validity of the random split.

### 3.2 Model development and comparison

**Figure 2:**
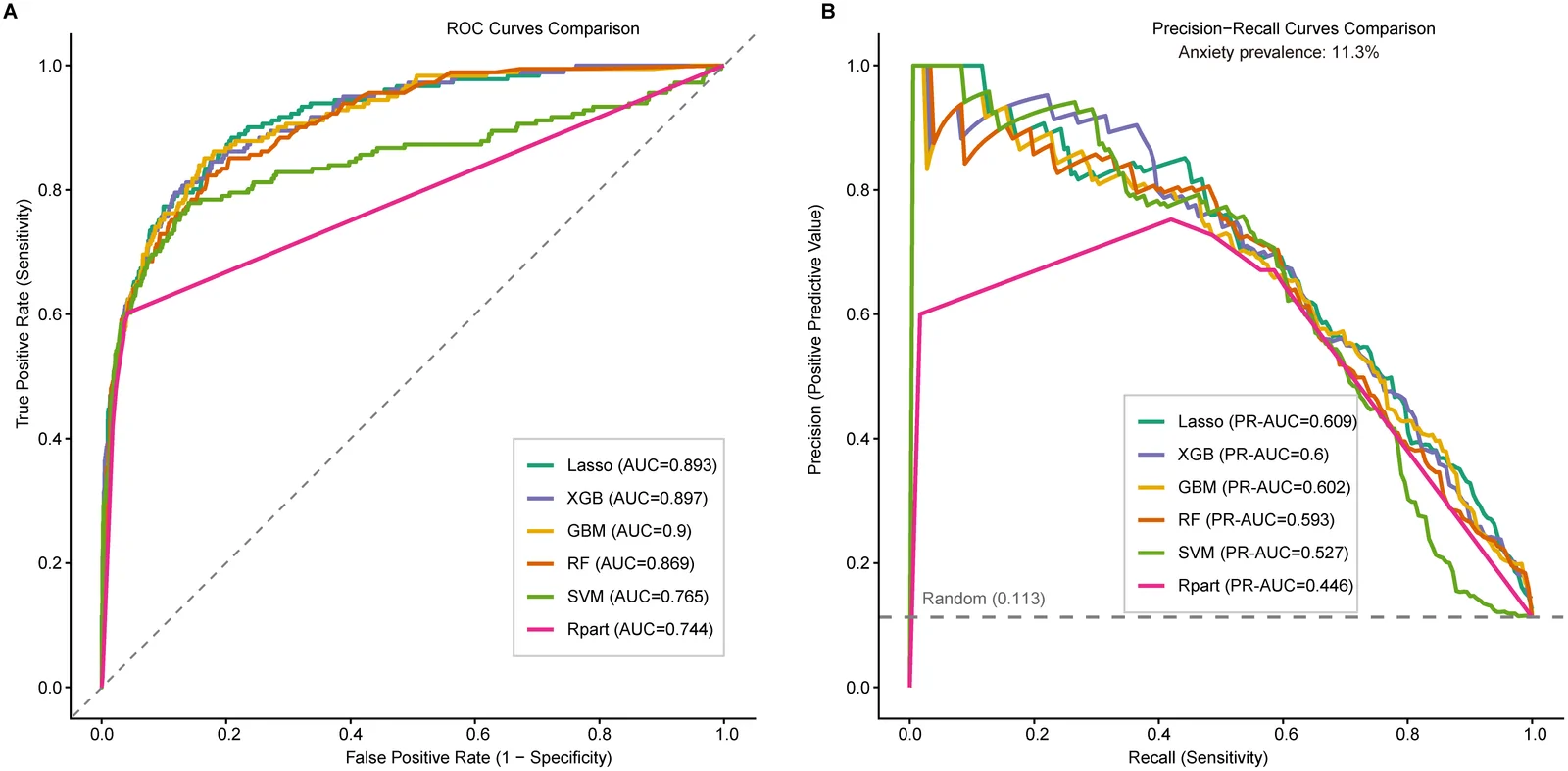
Performance of machine learning models for identifying anxiety symptoms. **(A)** ROC curves of the six ML models. **(B)** Precision-recall curves of the six ML models.

**Figure 3:**
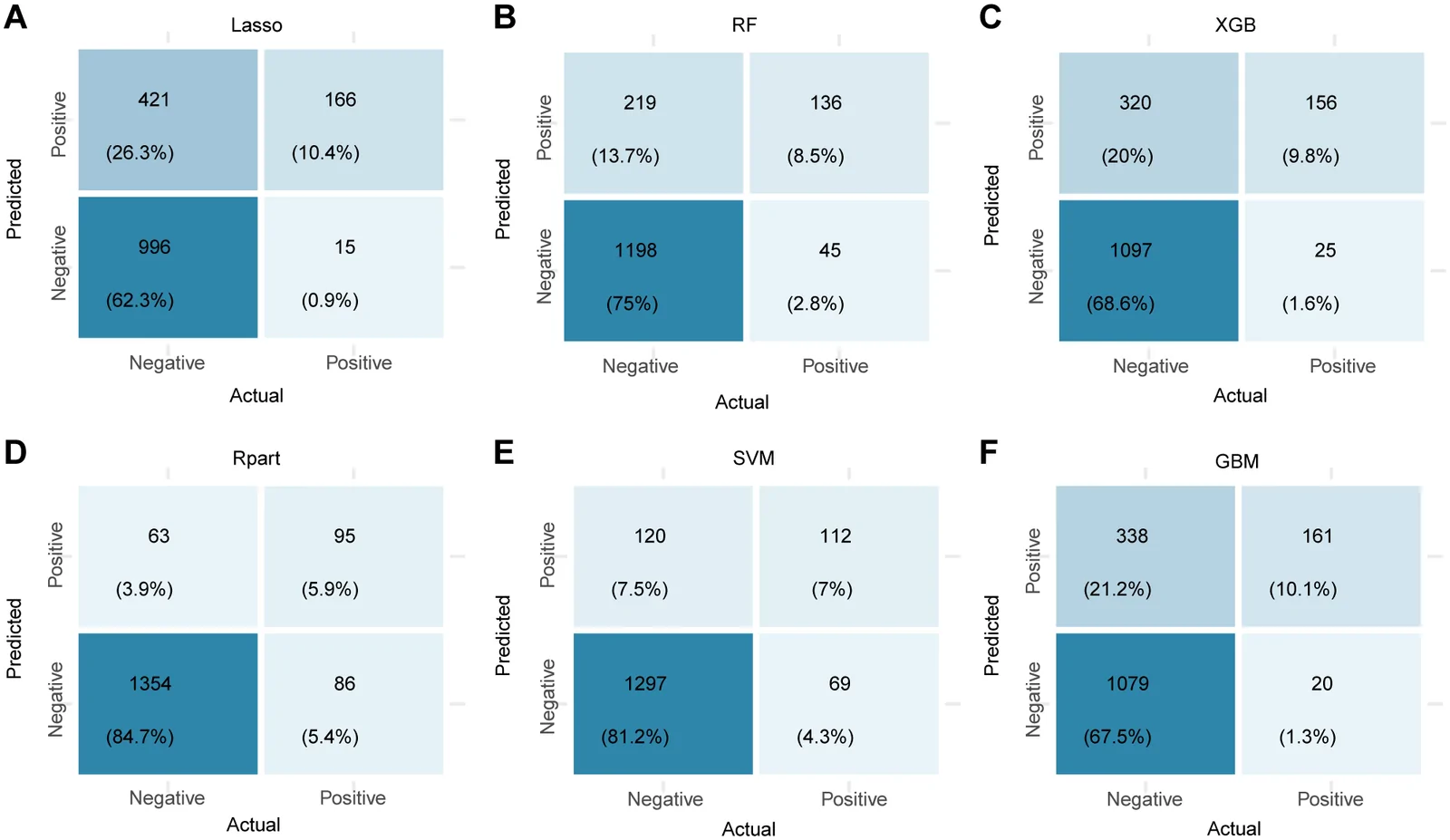
Confusion matrices of the six machine learning models on the test set. **(A)** Lasso. **(B)** Random Forest (RF). **(C)** XGBoost (XGB). **(D)** Decision tree (Rpart). **(E)** Support Vector Machine (SVM). **(F)** Gradient boosting machine (GBM).

This study adopted a structured model training and evaluation pipeline. The dataset was randomly split into training (70%, n = 3,733) and test sets (30%, n = 1,598) using stratified sampling. No oversampling technique (e.g., SMOTE) was applied to the training set, as we aimed to evaluate model performance under the natural class distribution (anxiety prevalence: 11.3%). The impact of different probability thresholds on performance metrics was examined *post-hoc*. Hyperparameters were optimized via grid search with 5-fold cross-validation on the training set. Six algorithms were developed: Lasso, Random Forest (RF), XGBoost (XGB), Decision Tree (Rpart), Support Vector Machine (SVM), and Gradient Boosting Machine (GBM). Their ROC curves and confusion matrices are presented in **Figures 2**, **3**, with performance metrics in **Table 3**.

**Table 3:** Performance metrics of six machine learning models on the test set.

Model	AUC	Accuracy	Sensitivity	Specificity	PPV	NPV	F1	Youden’s J
GBM	**0.8995**	0.7760	0.8895	0.7615	0.3226	0.9818	0.4735	**0.6510**
XGBoost	0.8967	0.7841	0.8619	0.7742	0.3277	0.9777	0.4749	0.6360
Lasso	0.8933	0.7272	**0.9171**	0.7029	0.2828	**0.9852**	0.4323	0.6200
Random Forest	0.8688	0.8348	0.7514	0.8454	0.3831	0.9638	0.5075	0.5968
SVM	0.7652	0.8817	0.6188	0.9153	0.4828	0.9495	0.5424	0.5341
Decision Tree	0.7442	**0.9068**	0.5249	**0.9555**	**0.6013**	0.9403	**0.5605**	0.4804

PPV, Positive Predictive Value; NPV, Negative Predictive Value. GBM, Gradient Boosting Machine; XGBoost, Extreme Gradient Boosting; SVM, Support Vector Machine. Best values per metric are highlighted in bold.

GBM achieved the highest AUC (0.8995) and sensitivity (0.8895), followed by XGB (AUC = 0.8967) and Lasso (AUC = 0.8933). For imbalanced data, PR-AUC is more informative than AUC; Lasso performed best (0.613), followed by GBM (0.606) and XGB (0.604), all substantially above the baseline prevalence (0.113). This suggests Lasso’s advantage in rare event detection despite its slightly lower AUC compared to GBM. Notably, both Lasso (sensitivity = 0.9171) and GBM (0.8895) correctly identified over 89% of anxious individuals.

**Table 4** presents DeLong test results. GBM, XGB, and Lasso showed no significant differences among themselves (all P > 0.05), but all three significantly outperformed RF, SVM, and Rpart (all P < 0.001).

**Table 4:** DeLong test for pairwise AUC comparisons.

Model 1	Model 2	AUC^1^	AUC^2^	P value	Significant
Lasso	XGBoost	0.8933	0.8967	0.5159	No
Lasso	GBM	0.8933	0.8995	0.2212	No
XGBoost	GBM	0.8967	0.8995	0.2512	No
Lasso	Random Forest	0.8933	0.8688	0.0012	Yes
Lasso	Rpart	0.8933	0.7442	<0.0001	Yes
Lasso	SVM	0.8933	0.7652	<0.0001	Yes
Random Forest	XGBoost	0.8688	0.8967	<0.0001	Yes
Random Forest	GBM	0.8688	0.8995	<0.0001	Yes
Random Forest	Rpart	0.8688	0.7442	<0.0001	Yes
Random Forest	SVM	0.8688	0.7652	<0.0001	Yes
XGBoost	Rpart	0.8967	0.7442	<0.0001	Yes
XGBoost	SVM	0.8967	0.7652	<0.0001	Yes
GBM	Rpart	0.8995	0.7442	<0.0001	Yes
GBM	SVM	0.8995	0.7652	<0.0001	Yes
Rpart	SVM	0.7442	0.7652	0.2528	No

AUC, area under the curve; GBM, Gradient Boosting Machine; XGBoost, Extreme Gradient Boosting; SVM, Support Vector Machine; RF, Random Forest; Rpart, Decision Tree.

### 3.3 Optimal model

**Figure 4:**
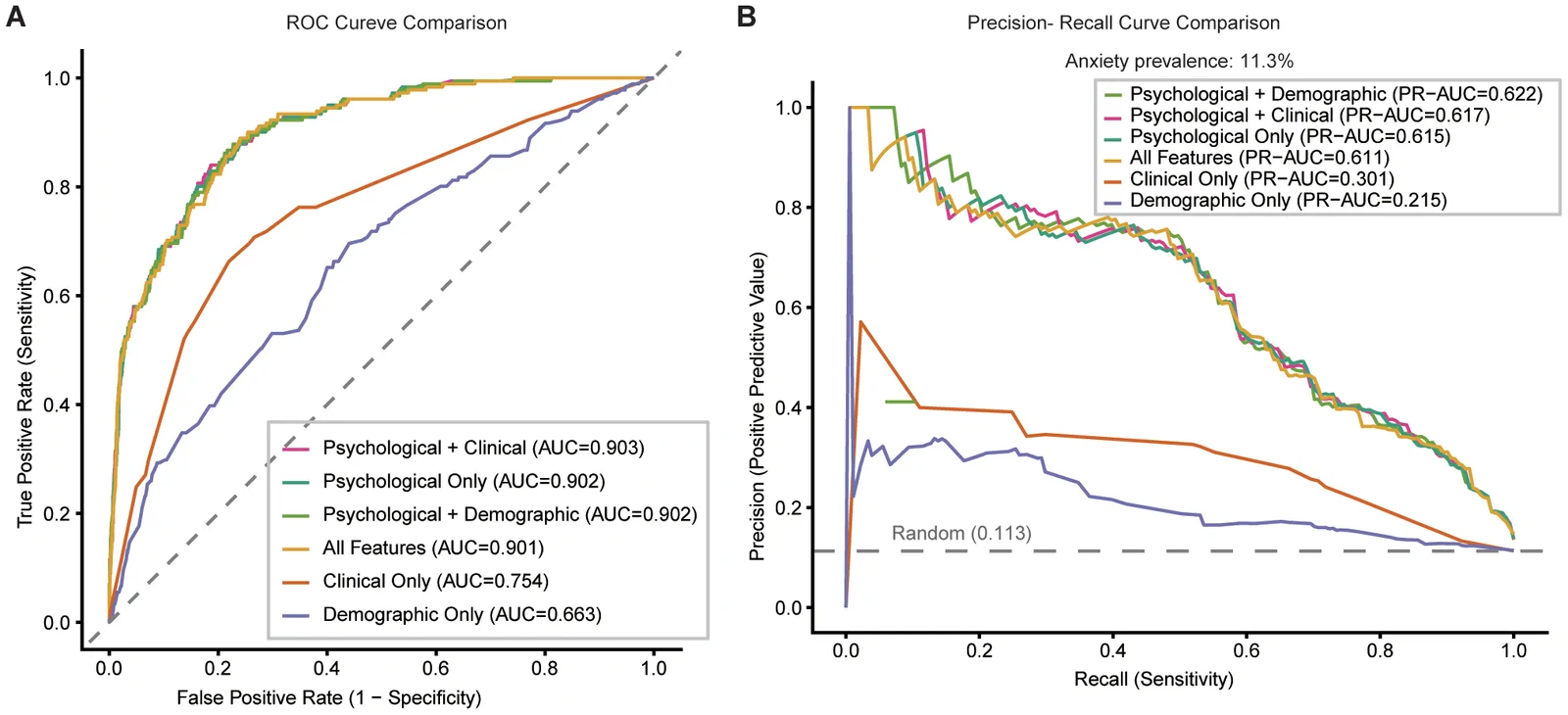
Performance of GBM model with different feature combinations. **(A)** ROC curves of GBM. **(B)** PRC curves of GBM.

Among the six models, GBM achieved the highest AUC (0.8995) and sensitivity (0.8895), while Lasso obtained the highest PR-AUC (0.613). DeLong tests showed no significant differences among GBM, XGBoost, and Lasso (all P > 0.05). Feature combination analysis revealed that the “Psychological + Clinical” set performed best (AUC = 0.9028, PR-AUC = 0.6173), as shown in **Table 5** and **Figure 4**. Therefore, GBM showed comparable performance to XGBoost and Lasso, with no statistically significant differences among them (all P > 0.05, DeLong test). Given its slightly higher AUC and sensitivity, GBM was selected for subsequent SHAP analysis and nomogram development.

**Table 5:** Comparison of GBM model performance across different feature combinations.

Combination	Youden_J	Threshold	Accuracy	F1	Sensitivity	Specificity	PPV	NPV	AUC	PR_AUC
Psychological + Clinical	0.6535	0.0696	0.8166	0.5092	0.8398	0.8137	0.3654	0.9755	0.9028	0.6173
Psychological Only	0.6438	0.0714	0.8166	0.5059	0.8287	0.8151	0.3641	0.9739	0.9024	0.6145
Psychological + Demographic	0.6512	0.0551	0.7847	0.4804	0.8785	0.7728	0.3306	0.9803	0.9018	0.6215
All Features	0.6496	0.0492	0.7747	0.4721	0.8895	0.7601	0.3214	0.9818	0.9012	0.6112
Clinical Only	0.4435	0.1529	0.7672	0.3922	0.663	0.7805	0.2784	0.9477	0.7539	0.3012
Demographic Only	0.2558	0.1166	0.5751	0.2707	0.6961	0.5596	0.168	0.9351	0.6629	0.2148

**Figure 5:**
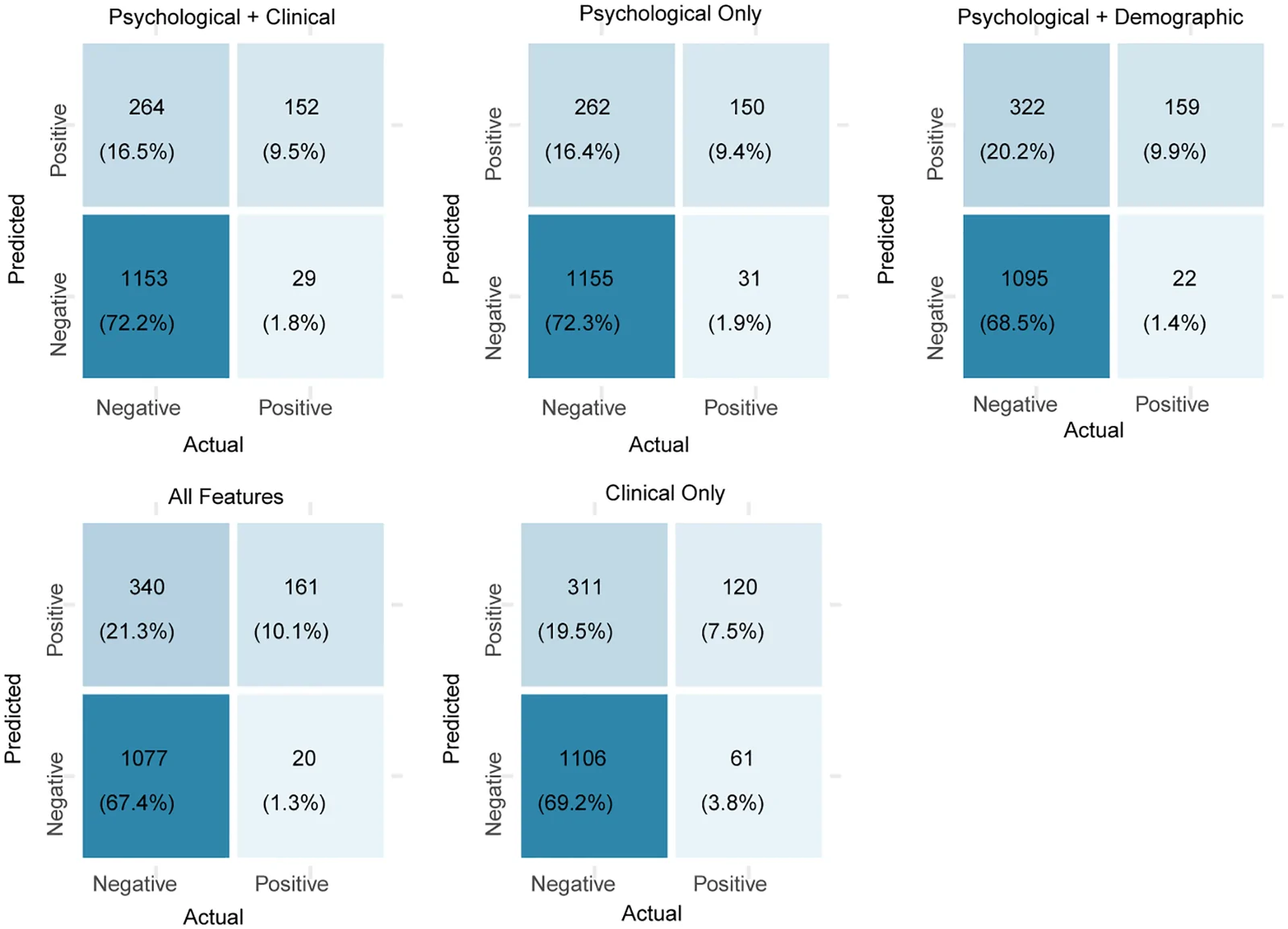
Confusion matrices of the GBM model with different feature combinations: psychological + clinical, psychological only, psychological + demographic, all features, and clinical only.

On the test set (**Table 5**), the optimal model achieved an AUC of 0.9028 and PR-AUC of 0.6173—well above the baseline prevalence (0.113). Sensitivity was 0.8398, specificity 0.8137, and NPV 0.9755. The confusion matrix (**Figure 5**) showed 152 true positives, 1,153 true negatives, 29 false negatives, and 264 false positives.

**Figure 6:**
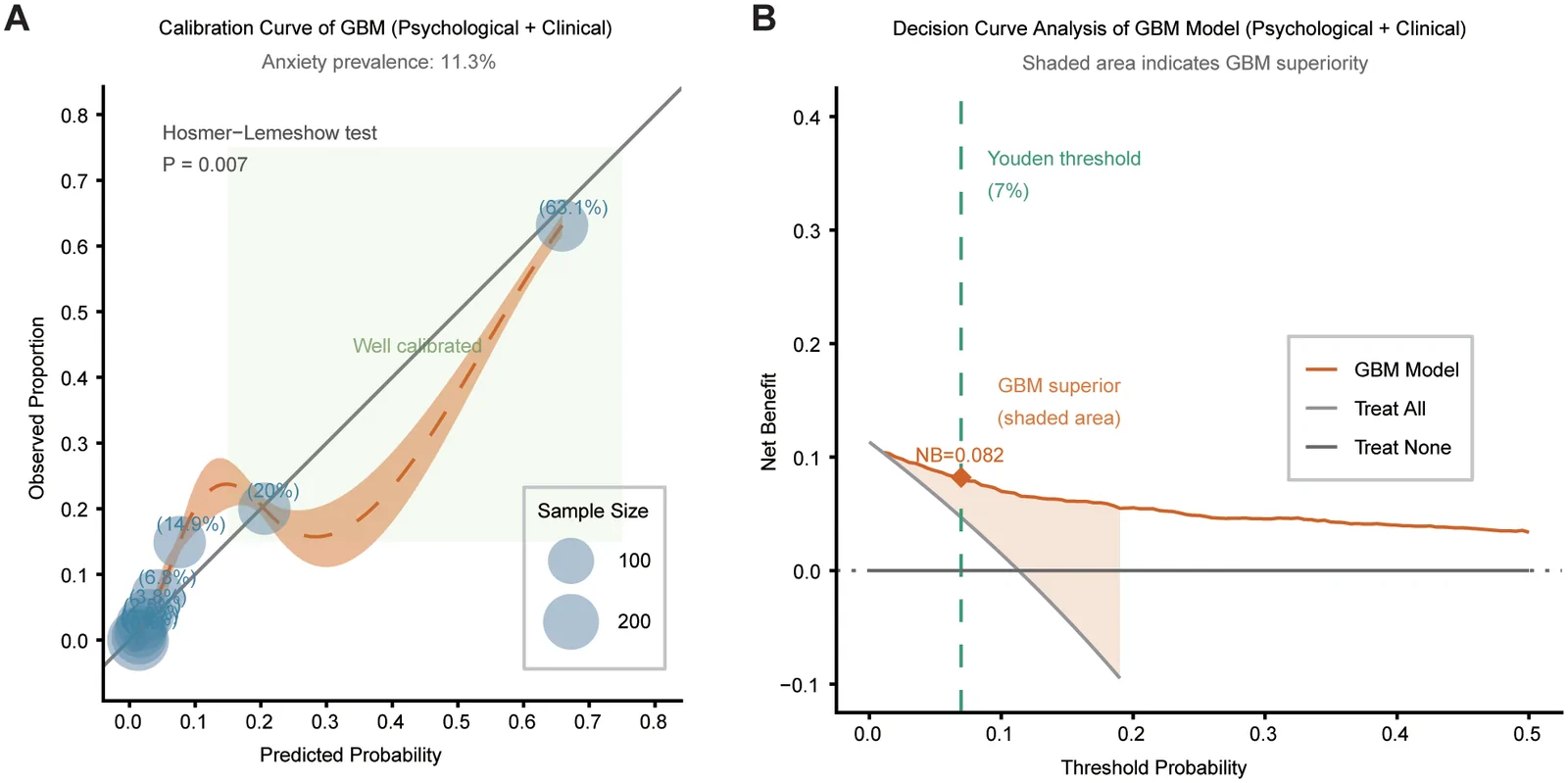
Model calibration and clinical utility assessment. **(A)** Calibration curve of the GBM model on the test set. **(B)** Decision curve analysis (DCA) of the GBM model.

Calibration analysis (**Figure 6A**) showed excellent agreement in the high-probability range (>20%) but mild overestimation at low risk (<5%). The Brier score was 0.059, and the calibration intercept (−0.077, P = 0.550) and slope (0.989, P = 0.851) indicated no significant miscalibration. Decision curve analysis (**Figure 6B**) demonstrated positive net benefit across thresholds of 5–35%, supporting the model’s clinical utility for anxiety screening in older adults.

### 3.4 Model explainability

Given the symptom overlap between depression and anxiety, we examined whether PHQ-9 dominance in SHAP analysis reflected genuine comorbidity or measurement artifact. PHQ-9 and GAD-7 were moderately-to-strongly correlated in the full sample (Spearman ρ = 0.618, 95% CI: 0.602–0.634), but this correlation attenuated substantially in the non-anxiety group (ρ = 0.37) compared with the anxiety group (ρ = 0.41). This attenuation is consistent with restriction of range due to dichotomization of GAD-7, which mechanically truncates variance within each subgroup, rather than reflecting Simpson’s paradox. This pattern supports discriminant validity in older adults without clinically significant anxiety, suggesting that PHQ-9’s high SHAP importance primarily reflects genuine depressive symptomatology rather than mere scale overlap.

**Figure 7:**
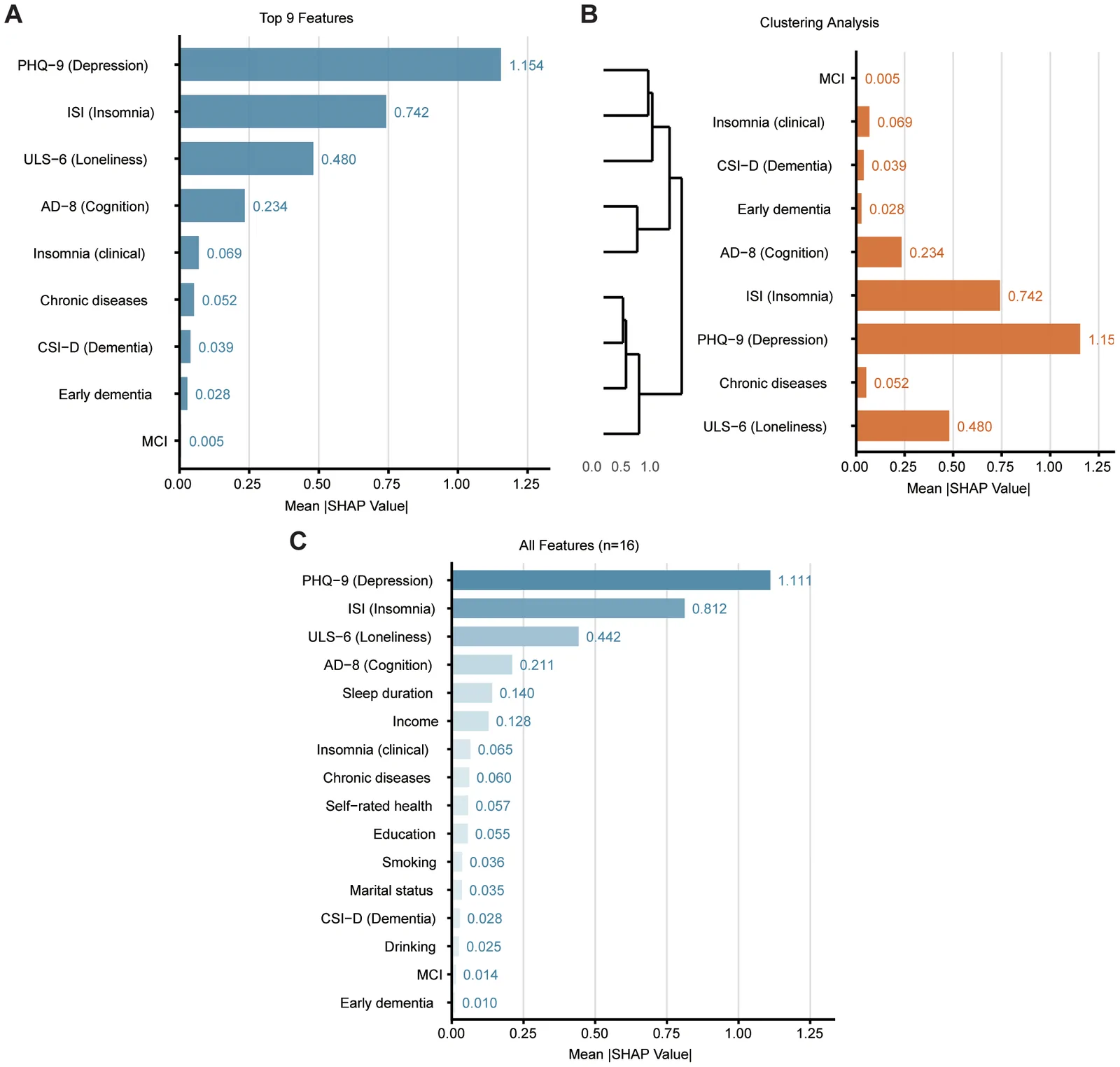
SHAP feature importance. **(A)** Bar chart of the top 9 features. **(B)** Bar chart with clustering analysis. **(C)** Bar chart of the all features.

SHAP analysis quantified each feature’s contribution to the GBM model. **Figure 7A** shows that PHQ-9 (depression) and ISI (insomnia) were the dominant predictors (mean SHAP = 1.154 and 0.742), followed by ULS-6 (loneliness, 0.480) and AD-8 (cognition, 0.234). The top four psychological scales accounted for 78.3% of the total predictive signal. **Figure 7B** presents clustering analysis, revealing two distinct clusters: psychological distress (PHQ-9, ISI, ULS-6) and cognitive impairment (AD-8, CSI-D, MCI, Dementia). **Figure 7C** confirms that the “Psychological + Clinical” combination captured most predictive signal, with marginal returns from additional variables.

**Figure 8:**
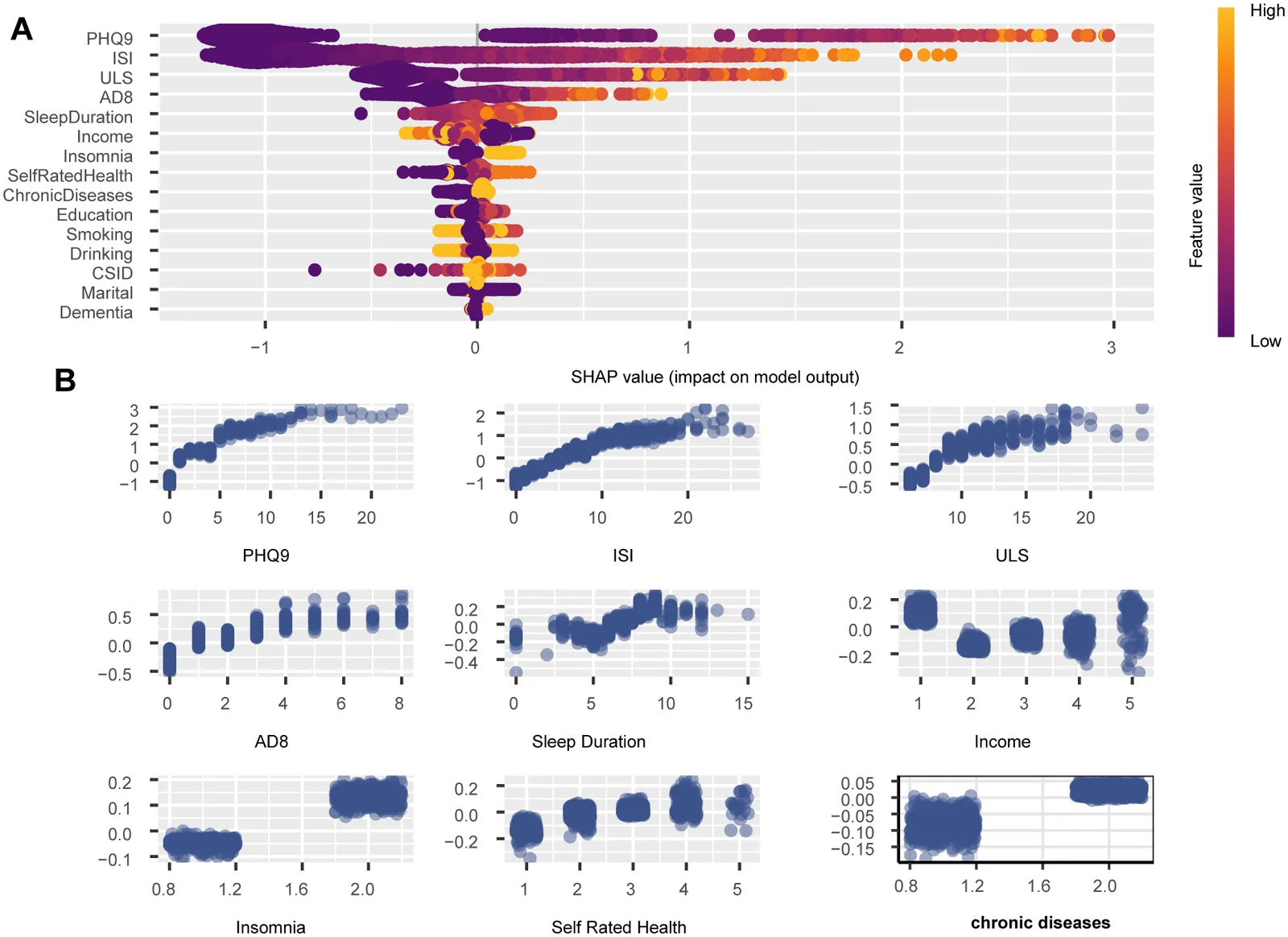
Global model explanation by the SHAP method. **(A)** Beeswarm plot. **(B)** Scatter plot.

**Figure 8A** (beeswarm plot) shows that higher PHQ-9, ISI, and ULS-6 scores (pink) consistently increased anxiety risk, while lower scores (blue) were protective. Demographic variables showed minimal impact. **Figure 8B** (dependence plots) reveals a near-linear relationship for PHQ-9, a steeper slope for ISI at scores >14, and a gradual rise for ULS-6.

**Figure 9:**
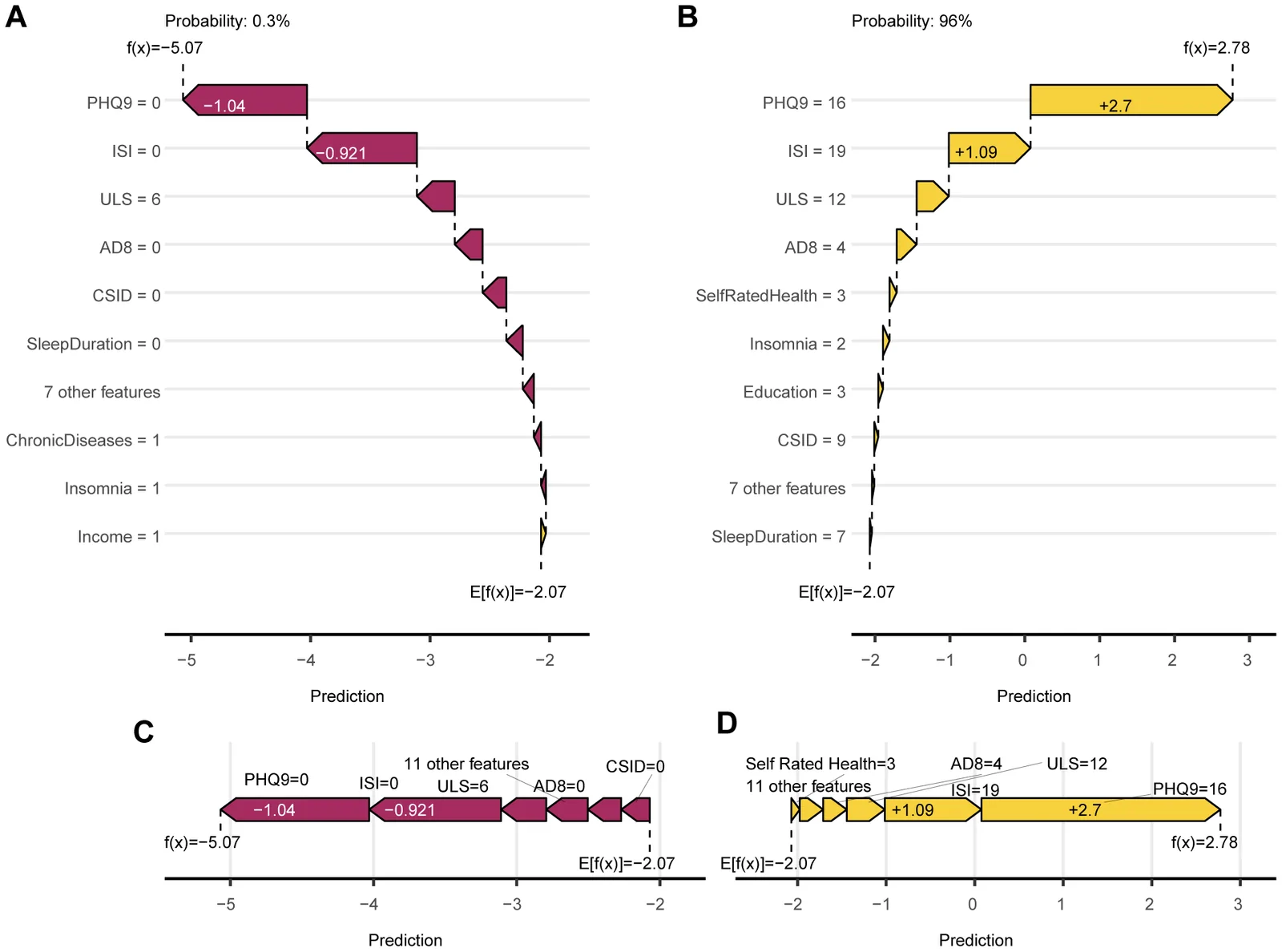
Local model explanation by the SHAP method. **(A)** Waterfall plot for a non-anxiety patient. **(B)** Waterfall plot for an anxiety patient. **(C)** Force plot for a non-anxiety patient. **(D)** Force plot for an anxiety patient.

**Figure 9** presents individualized explanations. For a non-anxiety patient (predicted risk: 3.2%), low PHQ-9 and absent insomnia were protective. For an anxiety patient (87.6%), severe PHQ-9 elevation and insomnia were the primary drivers. These results confirm that depression and insomnia are the principal levers for anxiety identification, enhancing clinical interpretability for targeted screening.

To evaluate the robustness of the model to predictor overlap, we conducted sensitivity analyses by re-training the GBM model after sequentially removing overlapping psychological scales. After excluding PHQ-9 alone, the model retained an AUC of 0.867 and PR-AUC of 0.494. After excluding both PHQ-9 and ISI, the model still achieved an AUC of 0.859 and PR-AUC of 0.463 (**Supplementary Table 1**). Even after removing three psychological scales (PHQ-9, ISI, and ULS-6), the AUC remained at 0.839, well above the baseline prevalence of 0.113. These results suggest that while depression-related symptoms contribute substantially to model performance, meaningful anxiety-relevant variance is captured by other features.

### 3.5 Subgroup and sensitivity analyses

**Figure 10:**
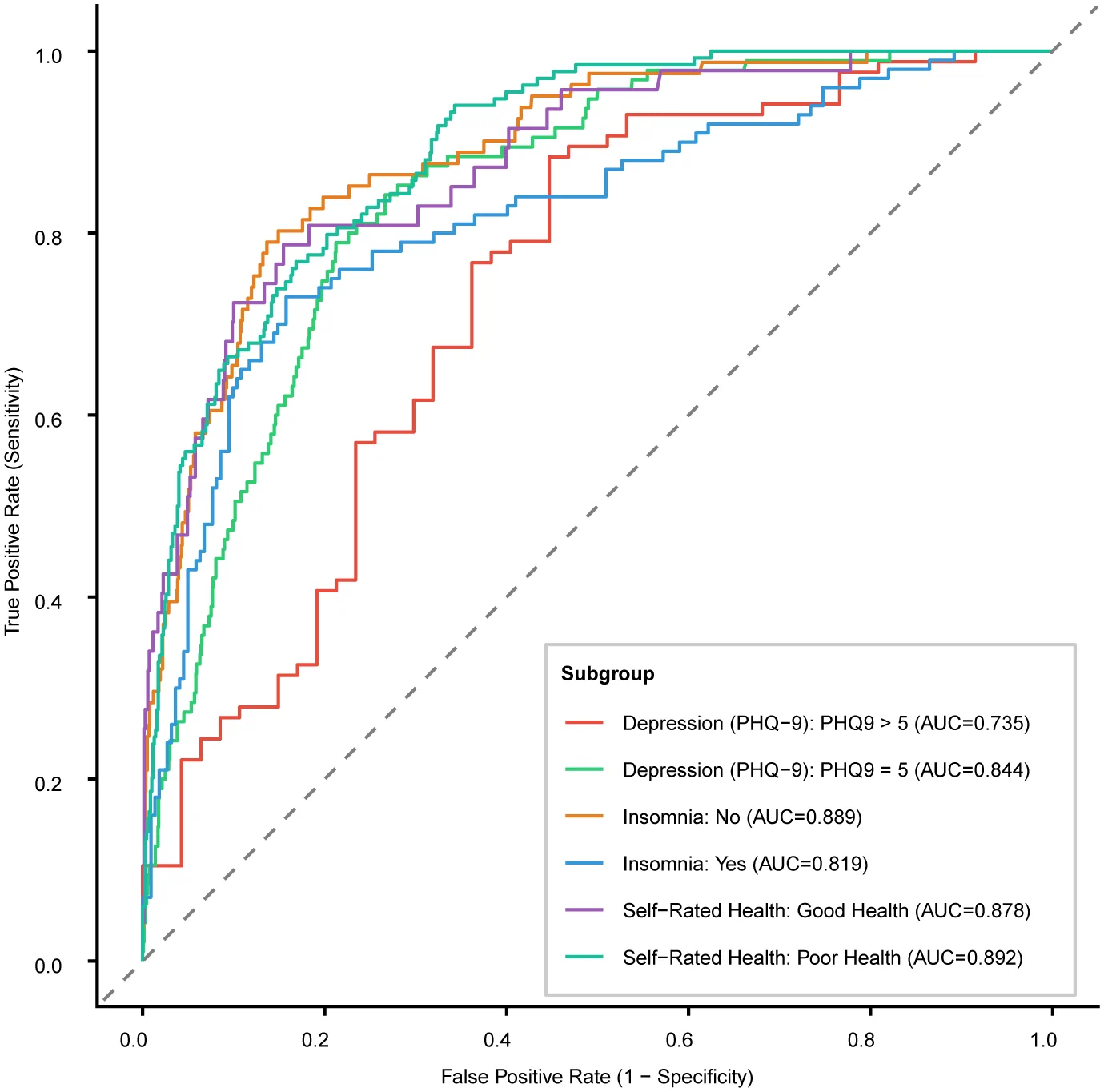
ROC curves of the GBM model by key subgroups.

Subgroup analyses were conducted to evaluate the model’s generalizability across different population strata (**Table 6**). The model performed notably well among current smokers (AUC = 0.966, 95% CI: 0.927–0.992; n = 195), participants with former drinking history (AUC = 0.935, 95% CI: 0.889–0.976; n = 150), and those with higher education (AUC = 0.945, 95% CI: 0.909–0.974; n = 212). However, some subgroups had limited sample sizes (e.g., ISI ≥ 14, n = 100; ULS ≥ 18, n = 23), and their AUC estimates have wide confidence intervals that should be interpreted with caution. Among sociodemographic strata, participants with higher education (AUC = 0.945, 95% CI: 0.909-0.974) and higher income (AUC = 0.934, 95% CI: 0.898-0.959) showed excellent discriminative performance. The model maintained good performance across most clinical subgroups, including those with chronic diseases (AUC = 0.899), without MCI (AUC = 0.907), and without dementia (AUC = 0.893). Notably, the model performed better among participants without insomnia (AUC = 0.890) than those with insomnia (AUC = 0.826), and substantially better among those with PHQ-9 <5 (AUC = 0.840) than those with PHQ-9 ≥5 (AUC = 0.750). **Figure 10** shows ROC curves of the GBM model by key subgroups (depression, insomnia, and self rate health), all demonstrating acceptable discriminative ability. As a sensitivity analysis, we re-ran the GBM model using a higher GAD-7 cutoff (≥10, indicating moderate-to-severe anxiety). At this threshold (prevalence = 2.2%), the model achieved an AUC of 0.900 (PR-AUC = 0.352, sensitivity = 0.743, specificity = 0.914, NPV = 0.994), confirming robustness across different outcome definitions.

**Table 6:** Subgroup analysis of GBM model performance on the test set.

Subgroup	Category	N	AUC	AUC_CI_Lower	AUC_CI_Upper	Accuracy	Sensitivity	Specificity
Education Level	High Education	515	0.929	0.902	0.96	0.866	0.81	0.874
Education Level	Low Education	1083	0.884	0.853	0.915	0.848	0.703	0.865
Marital Status	Unmarried/Divorced/Widowed	1313	0.908	0.886	0.928	0.856	0.744	0.871
Marital Status	Married/Cohabiting	285	0.863	0.75	0.916	0.846	0.72	0.858
Self-rated Health	Poor Health	840	0.892	0.86	0.912	0.824	0.754	0.837
Self-rated Health	Good Health	758	0.878	0.831	0.927	0.914	0.574	0.937

AUC, area under the curve; CI, confidence interval; GBM, Gradient Boosting Machine; PHQ-9, Patient Health Questionnaire-9.

Sensitivity analyses assessed the impact of different probability thresholds on model performance (**Table 1**). As the threshold increased from 0.03 to 0.10, sensitivity decreased from 0.934 to 0.746, while specificity improved from 0.629 to 0.856. The PPV increased modestly from 0.244 to 0.398, whereas NPV remained consistently high (>0.96 across all thresholds). These results highlight the trade-off between sensitivity and specificity, allowing clinicians to select thresholds based on screening objectives: lower thresholds (e.g., 0.03-0.05) prioritize sensitivity to minimize missed cases, while higher thresholds (e.g., 0.10) improve specificity to reduce false positives.

### 3.6 Clinical application

**Figure 11:**
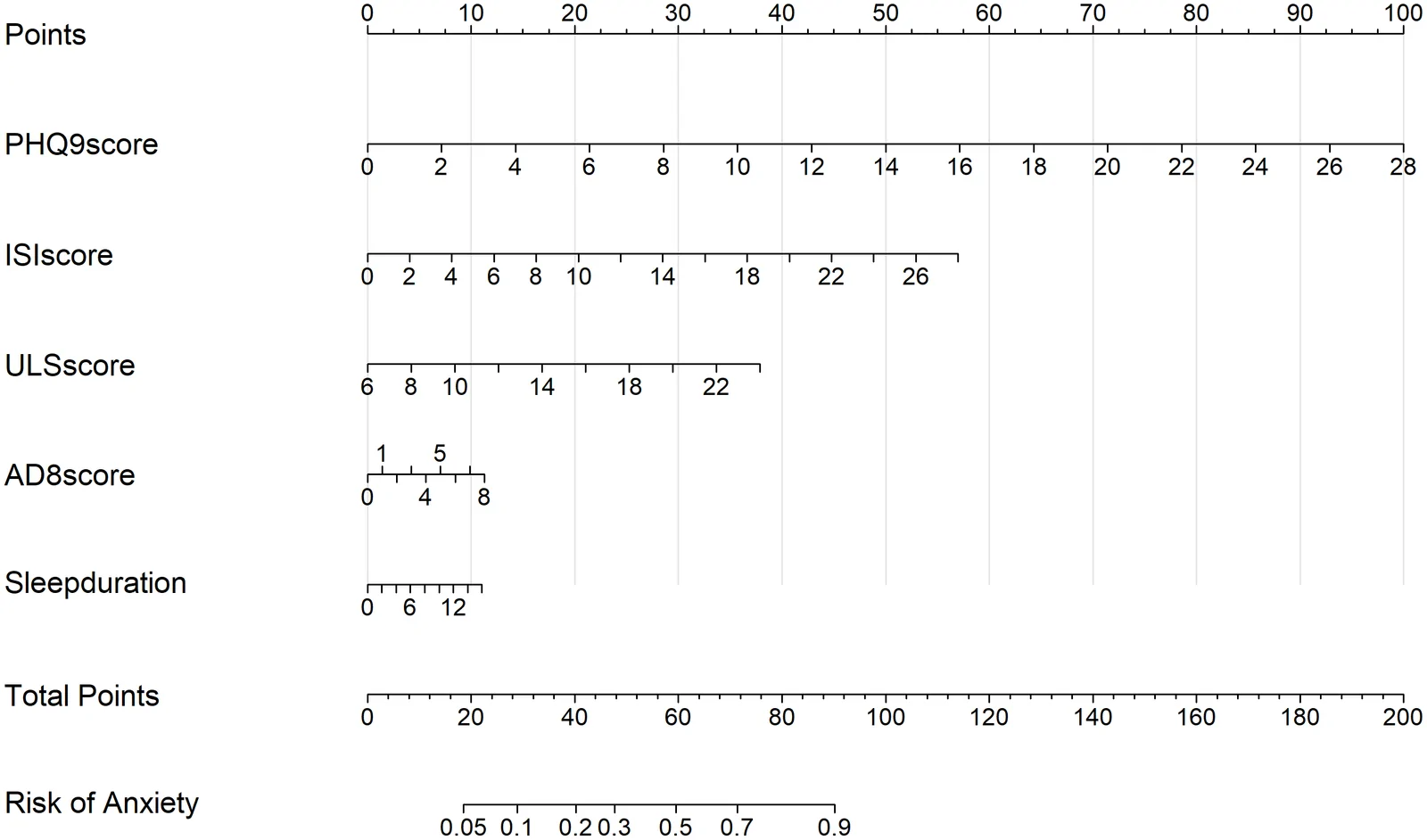
Nomogram for identifying anxiety risk in older adults. PHQ-9, Patient Health Questionnaire-9 (depression); ISI, Insomnia Severity Index; ULS-6, 6-item UCLA Loneliness Scale; AD-8, Ascertain Dementia Questionnaire-8 (cognitive decline).

A nomogram was developed based on a logistic regression model incorporating the five most important features identified by SHAP analysis: PHQ-9, ISI, ULS-6, AD-8, and sleep duration (**Figure 11**). The logistic model achieved a C-statistic of 0.916 (95% CI: 0.899–0.933) on the test set. To use the nomogram, locate each patient’s value on the corresponding variable axis, draw a line upward to the “Points” axis to assign points, sum all points to obtain the total score, and draw a line downward to the “Risk of Anxiety” axis to obtain the predicted probability. The regression coefficients and a simplified risk score sheet are presented in **Supplementary Table 2**.

## 4 Discussion

This study developed and systematically compared six machine learning algorithms for identifying anxiety symptoms in 5,331 community-dwelling older adults from Shenzhen, China. This study applies SHAP-based interpretable machine learning with coupled with a logistic regression-based nomogram for anxiety identification in Chinese older adults using a large community-based sample. Our main findings are threefold: 1) GBM achieved the best discriminative performance (AUC = 0.900), comparable to XGBoost and Lasso; 2) psychological factors—particularly depressive symptoms (PHQ-9), insomnia (ISI), and loneliness (ULS-6)—emerged as the dominant risk indicators; and 3) the developed nomogram and risk score sheet provide a practical, non-invasive tool for community-based anxiety screening.

The prevalence of anxiety symptoms in our sample was 11.3%, consistent with previous community-based studies in Chinese older adults (range: 8.5% to 15.2%) (2, 3). This figure is notably higher than the 5.97% 12-month prevalence reported by the China Mental Health Survey for adults aged 55 years and above (2), likely reflecting the inclusion of subthreshold anxiety (GAD-7 ≥ 5) rather than only full diagnostic criteria, as well as potential pandemic-related mental health effects given data collection occurred during 2020-2021 (1).

### 4.1 Model performance and algorithm selection

Among the six algorithms evaluated, GBM achieved the highest AUC (0.900), followed closely by XGBoost (0.897) and Lasso (0.893), with DeLong tests confirming no statistically significant differences among these three top performers (all P > 0.05). This finding aligns with recent comparative studies in psychiatric risk classification. A 2025 study comparing ML algorithms for depression prediction in older adults also found that gradient boosting methods (XGBoost and LightGBM) outperformed traditional logistic regression, though the performance gap was narrower than typically assumed (24). Similarly, a systematic review of ML-based anxiety prediction reported that ensemble methods consistently achieved AUCs between 0.85-0.92 across diverse populations, with gradient boosting showing particular advantages in handling nonlinear relationships and feature interactions (25).

The comparable performance of Lasso—a simpler, more interpretable linear model—with GBM and XGBoost is noteworthy. This finding suggests that for anxiety identification using self-reported psychological scales, the underlying signal may be sufficiently captured by linear relationships, reducing the need for complex ensemble architectures (6). This has practical implications for clinical implementation, as simpler models are easier to validate, maintain, and explain to patients and providers (26).

### 4.2 Key risk indicators: depression, insomnia, and loneliness

PHQ-9 emerged as the strongest predictor, consistent with established depression-anxiety comorbidity and supported by discriminant validity evidence. SHAP analysis identified PHQ-9 (depressive symptoms) as the single most important predictor, followed by ISI (insomnia) and ULS-6 (loneliness). The dominant role of depression in identifying anxiety is well-established, with meta-analytic evidence confirming a bidirectional relationship and 50-70% comorbidity between these conditions in older adults (27). Neurobiologically, both disorders share dysregulation of the serotonin and norepinephrine systems, as well as hypothalamic-pituitary-adrenal (HPA) axis hyperactivity (28, 29). Chronic stress drives HPA axis hyperactivity, elevating cortisol levels and causing hippocampal atrophy, prefrontal cortex dysfunction, and amygdala hyperactivity—key contributors to emotional dysregulation in both depression and anxiety (30, 31).

Insomnia emerged as the second most important predictor, consistent with extensive literature documenting the bidirectional relationship between sleep disturbance and anxiety. A 2024 meta-analysis found that insomnia increased the risk of incident anxiety in older adults (32). The relationship is mechanistically plausible: sleep deprivation impairs emotion regulation by reducing prefrontal cortical control over the amygdala, while anxiety-related hyperarousal perpetuates sleep maintenance difficulties (33). Notably, our SHAP dependence plot for ISI showed a steeper slope at scores >14, suggesting a potential threshold effect where severe insomnia disproportionately amplifies anxiety risk—a finding that may inform treatment prioritization in clinical practice.

Loneliness ranked third, reflecting growing recognition of social isolation as a key determinant of late-life mental health. A 2023 meta-analysis of 90 cohort studies reported that loneliness increased the risk of anxiety disorders by 1.8-fold in older adults (34). The COVID-19 pandemic likely exacerbated this relationship, given that our data collection coincided with periods of social distancing (35, 36). Uniquely, loneliness may be particularly modifiable through community-based interventions (e.g., social prescribing, group activities), suggesting actionable targets for anxiety prevention (37).

A potential concern is whether the model merely detects general psychological distress rather than anxiety-specific symptoms, given the strong contribution of PHQ-9. However, our sensitivity analysis showed that the model retained acceptable discrimination (AUC = 0.859) even after excluding both PHQ-9 and ISI, suggesting that other features—including loneliness, cognitive function, and clinical conditions—capture meaningful anxiety-relevant variance beyond shared distress. Nevertheless, we acknowledge that depression-anxiety comorbidity is intrinsic to late-life mental health, with meta-analyses reporting comorbidity rates of 40–60% in older adults, and that complete separation of these constructs may not be clinically realistic or desirable. Furthermore, as all variables were self-reported and collected simultaneously, shared method variance may have inflated the observed associations between psychological scales and anxiety status.

### 4.3 Feature combination analysis

We systematically evaluated six feature combinations and found that the “Psychological + Clinical” set achieved the best overall performance (AUC = 0.903). Notably, psychological scales alone performed nearly as well (AUC = 0.902), while adding demographic factors provided minimal incremental benefit. This finding has significant practical implications for community screening: a brief battery of five psychological scales (PHQ-9, ISI, ULS-6, AD-8, CSI-D)—which can be administered in approximately 10–15 minutes—may be sufficient for accurate anxiety risk stratification, without requiring extensive demographic or clinical data collection (38). This aligns with the principles of parsimonious screening and reduces burden on both older adults and primary care providers.

The marginal contribution of chronic diseases to model performance was unexpected, given established associations between multimorbidity and late-life anxiety (39, 40). However, our SHAP analysis revealed that the effect of chronic diseases was largely mediated through psychological scales, particularly PHQ-9 and ISI. This suggests that the relationship between physical illness and anxiety operates through symptom burden (depression, sleep disturbance, pain) rather than diagnosis labels per se—a finding consistent with the biopsychosocial model of illness (36).

### 4.4 Subgroup and sensitivity analyses

Subgroup analyses revealed excellent model performance across most population strata. The model performed best among current smokers and higher-income participants, though these subgroups had smaller sample sizes, warranting cautious interpretation. Notably, performance was lower among participants with PHQ-9 ≥5 compared to those without depression. This finding suggests that predicting anxiety becomes more challenging in the context of comorbid depression, likely due to symptom overlap and the difficulty of distinguishing between these conditions. Clinically, this implies that individuals with comorbid depression-anxiety may require more comprehensive assessment rather than relying solely on automated screening tools.

Sensitivity analyses examining threshold effects demonstrated the expected trade-off between sensitivity and specificity, allowing clinicians to select operating thresholds based on screening objectives. For population-based screening prioritizing case-finding (minimizing false negatives), a lower threshold would be appropriate; for confirmatory settings or when diagnostic resources are limited, a higher threshold may be preferred to reduce false positives (6). The consistently high NPV across all thresholds supports the model’s utility for ruling out anxiety—a clinically valuable property for primary care triage.

### 4.5 Comparison with previous ML identification studies

Our findings extend the growing literature on ML-based mental health identification in several important ways. First, most previous ML studies in anxiety identification have focused on Western populations, limiting generalizability to Chinese older adults given substantial cultural differences in symptom expression, help-seeking behaviors, and healthcare utilization (20, 35). Our study addresses this gap by developing and validating models specifically for a large Chinese community sample.

Second, unlike many previous studies that employed single algorithms or lacked interpretability features, our systematic comparison of six algorithms with SHAP analysis provides both performance benchmarking and mechanistic insights. This dual focus addresses a key limitation noted in recent systematic reviews, which have criticized the “black-box” nature of many clinical identification models as a barrier to clinical adoption (36). The presence of an interpretable nomogram and risk score sheet further enhances clinical utility.

### 4.6 Clinical implications

Our study has several practical implications for primary care and community mental health services. First, the logistic regression-based nomogram and point-based risk score sheet enable rapid, non-computational risk stratification. Given that primary care providers in China often lack specialized mental health training and face time constraints (41), a paper-based tool requiring only simple arithmetic has potential for clinical application following external validation. However, these implications are contingent upon successful external validation, and clinical deployment should be preceded by independent multi-center studies.

Second, the superior performance of the “Psychological + Clinical” combination suggests that community screening programs can focus on a brief set of psychological scales, rather than comprehensive clinical assessments (42). This reduces administration time and cost, facilitating implementation pending external validation, although large-scale implementation would require prior validation across diverse settings. Using conservative assumptions (10 minutes per administration), a single community health worker could screen approximately 48 older adults per day.

Third, the identification of modifiable risk factors—particularly insomnia and loneliness—provides clear targets for intervention. Anxiety intervention programs might prioritize sleep hygiene education (43) and social engagement initiatives (44), complementing individual-level screening with community-level prevention.

Fourth, the clinical utility of our model must be considered alongside its positive predictive value. At the observed anxiety prevalence of 11.3%, even an AUC of 0.90 yields a PPV of approximately 0.32–0.40, meaning that 60–68% of positive screens would be false positives—a well-known Bayesian consequence in low-prevalence settings and comparable to the performance of PHQ-9 and GAD-7 in primary care. The model’s primary strength therefore lies in its high NPV (0.98) for ruling out anxiety, and it should be viewed as a pre-screening tool rather than a diagnostic instrument. It is reasonable to ask why a clinician would administer multiple scales rather than a single brief scale such as the GAD-7. While a single scale efficiently identifies symptom severity, it does not discriminate between etiological drivers. Our model addresses this by providing a multi-dimensional risk profile that can guide personalized intervention—for example, distinguishing between anxiety driven by insomnia versus depressive symptoms. Nonetheless, we acknowledge the added assessment burden and suggest that future studies explore abbreviated screening batteries (42).

Fifth, several variables known to be associated with late-life anxiety—including physical activity, social participation, medication use, pain, functional status, frailty, and healthcare utilization—were unavailable in this dataset. Future studies incorporating these variables may further improve model performance, particularly for subgroups where physical frailty or chronic pain are primary drivers of anxiety.

### 4.7 Limitations

Several limitations should be acknowledged. First, the cross-sectional design precludes causal inference and therefore limits evaluation of the model’s performance for identifying individuals at risk of future anxiety; future prospective studies are needed to assess temporal identification. Second, all participants were recruited solely from Shenzhen, a highly developed coastal city, which may limit generalizability to older adults in rural areas or other Chinese provinces, and external validation in geographically diverse cohorts is necessary. Third, model performance was evaluated using only an internal test set without external validation, raising concerns about overfitting; moreover, the positive predictive value (PPV) was only 0.3226 at the optimal threshold, meaning approximately 68% of positive screens would be false positives—a limitation partly attributable to the low prevalence (11.3%)—so the model should be used as a pre-screening tool with confirmatory diagnostic interviews for positive screens. Fourth, the absence of missing data in this large community sample warrants cautious interpretation, as it may reflect exclusion of incomplete cases and potential selection bias.

Several limitations should be acknowledged. First, the cross-sectional design precludes causal inference and limits evaluation of temporal identification; prospective studies are needed to assess predictive performance over time. Second, all participants were recruited from Shenzhen, which may limit generalizability to other regions; external validation in geographically diverse cohorts is necessary. Third, several potentially important variables—including physical activity, social participation, medication use, pain, functional status, and frailty—were unavailable in this secondary dataset, and their omission may have contributed to residual confounding. Fourth, all measures were self-reported and collected simultaneously, which may introduce common method bias; future studies using clinician-administered assessments (e.g., MINI, SCID) and objective measurements (e.g., actigraphy) would strengthen the evidence. Fifth, the strong contribution of depression-related variables raises the possibility that the model partially detects general psychological distress rather than anxiety specifically, although sensitivity analyses excluding PHQ-9 and ISI suggest that meaningful variance beyond shared distress remains. Additionally, some subgroup analyses involved small samples (e.g., n = 23 for ULS ≥ 18), and these results should be considered exploratory.

## 5 Conclusion

This study demonstrated that machine learning models, particularly GBM, achieved excellent performance in identifying anxiety symptoms among Chinese community-dwelling older adults. SHAP analysis identified depressive symptoms, insomnia, and loneliness as the dominant risk indicators, while feature combination analysis revealed that psychological scales alone performed nearly as well as more complex combinations. The developed nomogram and point-based risk score sheet provide non-invasive screening tools. Given the modest PPV, the model is best suited as a pre-screening instrument to identify high-risk individuals for further evaluation, with its primary strength lying in its high NPV for ruling out anxiety. However, these findings are based on internal validation only. Future studies should validate the model in independent geographic cohorts and prospective settings before clinical translation.

## Data Availability

The original contributions presented in the study are included in the article/**Supplementary Material**. Further inquiries can be directed to the corresponding author.
