# Supplementary material for: Machine learning-based identification of anxiety symptoms in Chinese community-dwelling older adults: a comparative study of six algorithms with SHAP analysis and nomogram development
Source: Front Psychiatry. 2026 Aug 20;17:1874650. doi: 10.3389/fpsyt.2026.1874650 (PMC13540043; doi:10.3389/fpsyt.2026.1874650)
Supplement: Supplementary file 1 [file Table1.docx]

**Supplementary Tables**

**Supplementary Table S1**. GBM model performance after sequential exclusion of overlapping psychological scales.

| Feature_Set | AUC | PR_AUC | Sensitivity | Specificity | PPV | NPV |
| --- | --- | --- | --- | --- | --- | --- |
| Full Model (16 features) | 0.91 | 0.648 | 0.845 | 0.822 | 0.377 | 0.977 |
| Excluding PHQ-9 | 0.867 | 0.494 | 0.768 | 0.806 | 0.336 | 0.965 |
| Excluding PHQ-9 + ISI | 0.859 | 0.463 | 0.773 | 0.822 | 0.356 | 0.966 |
| Excluding PHQ-9 + ISI + ULS-6 | 0.839 | 0.443 | 0.834 | 0.71 | 0.269 | 0.971 |

Note: AUC, area under the curve; PR-AUC, precision-recall area under the curve; PPV, positive predictive value; NPV, negative predictive value. Baseline anxiety prevalence in the test set was 0.113. All models were trained using GBM with identical hyperparameters .

**Supplementary Table S2.** Logistic regression coefficients for the nomogram prediction model (top five SHAP-identified predictors).

| Predictor | Coefficient | OR | CI_95 | P_value |
| --- | --- | --- | --- | --- |
| Intercept | -4.9988 | 0.0067 | [0.0034, 0.0135] |  |
| PHQ-9 (Depression) | 0.2579 | 1.2942 | [1.2488, 1.3412] | 0 |
| ISI (Insomnia) | 0.1504 | 1.1622 | [1.1317, 1.1936] | 0 |
| ULS-6 (Loneliness) | 0.1365 | 1.1463 | [1.1049, 1.1892] | 0 |
| AD-8 (Cognition) | 0.0958 | 1.1005 | [1.0305, 1.1753] | 0.0043 |
| Sleep Duration (hours) | 0.0378 | 1.0386 | [0.9603, 1.1232] | 0.344 |

Note: OR, odds ratio; CI, confidence interval. The logistic regression model was fitted on the training set (N = 3,732) using the rms package in R. C-statistic on the test set = 0.916. Sleep duration was not statistically significant (P = 0.344) but was retained as it was among the top five SHAP-identified predictors.
